# Face Boundary Formulation for Harmonic Models: Face Image Resembling

**DOI:** 10.3390/jimaging11010014

**Published:** 2025-01-08

**Authors:** Hung-Tsai Huang, Zi-Cai Li, Yimin Wei, Ching Yee Suen

**Affiliations:** 1 Department of Data Science and Analytics, I-Shou University, Kaohsiung 84001, Taiwan; huanght@isu.edu.tw; 2 Department of Applied Mathematics, National Sun Yat-sen University, Kaohsiung 80424, Taiwan; zicaili1@gmail.com; 3 Shanghai Key Laboratory of Contemporary Applied Mathematics, Fudan University, Shanghai 200433, China; ymwei@fudan.edu.cn; 4 Center for Pattern Recognition and Machine Intelligence, Concordia University, Montreal, QC H3G 1M8, Canada

**Keywords:** face boundary formulation, blending curves, ODE using Hermite interpolation, splitting–integrating method, harmonic models, age effects, face combination and resembling, face morphing, morphing attack detection

## Abstract

This paper is devoted to numerical algorithms based on harmonic transformations with two goals: (1) face boundary formulation by blending techniques based on the known characteristic nodes and (2) some challenging examples of face resembling. The formulation of the face boundary is imperative for face recognition, transformation, and combination. Mapping between the source and target face boundaries with constituent pixels is explored by two approaches: cubic spline interpolation and ordinary differential equation (ODE) using Hermite interpolation. The ODE approach is more flexible and suitable for handling different boundary conditions, such as the clamped and simple support conditions. The intrinsic relations between the cubic spline and ODE methods are explored for different face boundaries, and their combinations are developed. Face combination and resembling are performed by employing blending curves for generating the face boundary, and face images are converted by numerical methods for harmonic models, such as the finite difference method (FDM), the finite element method (FEM) and the finite volume method (FVM) for harmonic models, and the splitting–integrating method (SIM) for the resampling of constituent pixels. For the second goal, the age effects of facial appearance are explored to discover that different ages of face images can be produced by integrating the photos and images of the old and the young. Then, the following challenging task is targeted. Based on the photos and images of parents and their children, can we obtain an integrated image to resemble his/her current image as closely as possible? Amazing examples of face combination and resembling are reported in this paper to give a positive answer. Furthermore, an optimal combination of face images of parents and their children in the least-squares sense is introduced to greatly facilitate face resembling. Face combination and resembling may also be used for plastic surgery, finding missing children, and identifying criminals. The boundary and numerical techniques of face images in this paper can be used not only for pattern recognition but also for face morphing, morphing attack detection (MAD), and computer animation as Sora to greatly enhance further developments in AI.

## 1. Introduction

### 1.1. Review of Previous Work

The face is the most prominent feature of a human being. Therefore, studies regarding face fusion and morphing remain active. In particular, face representation has been studied by Gabor wavelets in Du and Ward [1], and face recognition and ear recognition have been also reported by Chang et al. [2]. For face recognition and face morphing, we also cite some recent reports: Aloraibi [3], Indrawal and Sharma [4], Patel and Lapsiwala [5], Scherhag et al. [6,7], Tuncer et al. [8], Guo and Zhang [9], You et al. [10], and Venkatesh et al. [11]. This paper is a continued study of Li, Chiang, and Suen [12] who studied face image transformations using three steps of numerical techniques.

**Step** **I:** Find the face boundary.
**Step** **II:** Carry out harmonic transformations by FEM, FDM, FVM, etc.
**Step** **III:** Transmit facial grayness by splitting algorithms.

We have devoted great efforts to splitting algorithms in Step III (see [13,14]) and harmonic transformations by numerical methods in Step II (see [12]). This paper will focus on Step I, which is to find face boundaries and their conversions during harmonic transformations.

Numerical methods are the main tools to perform face image transformation. For image transformation, new numerical methods have been studied since Li et al. [14], and a number of papers have been published. Among them, the splitting–integrating method (SIM) and harmonic transformation are the most powerful and advanced. The boundary is most important in describing and recognizing the pattern, the individual person, and the transformed models. Harmonic transformations consist of a couple of Laplace’s equations in the face domains and face boundaries. In fact, once the interior and exterior boundaries of face images are known, face domains are defined. Moreover, for each person, the formulation of face boundaries in digital images may be discriminative, complicated, and laborious.

For the boundary of the harmonic model, we solicit the blending curves. Surfaces and curves identified by interpolation are well-known and described in many reports [15,16]. In this paper, new methods for blending curves will be explored for generating face boundaries based on ordinary differential equations (ODE) and their numerical solutions [17]. It is worth pointing out that since the publication of [14], the basic numerical methods for geometric image transformations have been studied and developed, and the face transformations in this paper and [12] are important applications and developments of our basic research. The image examples of face combinations and face resembling in this paper can also find applications in many areas, such as criminal identification, finding missing children, plastic surgery, marriage counseling by predicting possible offspring images, etc. Evidently, the numerical techniques of face boundary in this paper can be used not only for pattern recognition but also for face fusion, face resampling, imaging morphing, morphing attack detection (MAD), and computer animation as Sora. It is also worth pointing out that the numerical algorithms in this paper are beneficial not only to face transformations but also to image geometric transformations.

### 1.2. Outlines of Numerical Face Transformations

Consider the nonlinear transformation as the harmonic transformation in [14]:

(1) $$\begin{array}{ccc} & & \Omega \overset{T}{\to }S,\;\partial \Omega \overset{T}{\to }\partial S, \end{array}$$

where T:(ξ,η)→(x,y), and the harmonic functions

(2) $$\begin{array}{ccc} & & x=x(\xi ,\eta ),\;\;y=y(\xi ,\eta ) \end{array}$$

satisfy the Laplace equations with the following Dirichlet conditions:

(3) $$\begin{array}{cc} & \Delta x=\frac{\partial^{2}x}{\partial \xi^{2}}+\frac{\partial^{2}x}{\partial \eta^{2}}{=0,\;\;x|}_{\partial \Omega }=\bar{x}, \end{array}$$

(4) $$\begin{array}{cc} & \Delta y=\frac{\partial^{2}y}{\partial \xi^{2}}+\frac{\partial^{2}y}{\partial \eta^{2}}{=0,\;\;y|}_{\partial \Omega }=\bar{y}, \end{array}$$

and $\bar{x}$ and $\bar{y}$ are given. For Steps I–III, the detailed techniques are described in the following sections.

Step I: Generate blending curves for mapping source and target face boundaries.

Boundary images have been treated by segmentation techniques, where superquadric models are explored, and the simple boundary is studied in [18] by affine kernel transformations. Furthermore, the symmetry of the boundary was explored in [19], and the piecewise linear approximation was used for planar curves for pattern recognition. These studies are confined to some special and simple types of image boundaries. This paper will deal with a rather general boundary of face images.

Let us briefly address the ideas of the blending techniques used in this paper. Based on the scattered 2D points, formulating a smooth curve is one of the basic topics in numerical analysis. Interpolation techniques, such as the Lagrange, Hermite, and spline interpolations, can be used. However, in this paper, we mimic a thin and flexible beam to be blended to pass through these given points exactly. Such a blending beam can be described as an ODE of order four with suitable boundary conditions. We choose the piecewise cubic Hermite polynomials as the admissible functions for the fourth-order ODE. The advantages of this technique are as follows: (1) the global curvature of the blending curve is minimal, and (2) the blending boundary has continuous derivatives (i.e., continuous slopes), thus fitting better and more naturally to the face boundary. The blending techniques are used for shaped evolution with structural and topological changes in [20] to “glue” two or several cutting surfaces together as one uniform subject. In fact, traditional techniques of blending surfaces in [15,21] can also be employed. However, we may also solicit the numerical PDE of biharmonic equations to better “glue” the cutting surfaces. Details are omitted.

First, the following preliminary work is prepared:

(a) Input a source, which is a 2D face image.
(b) Choose a target face frame to be transformed (or resembled).
(c) Locate the control and characteristic points along open or closed curves on the face boundary and the important facial features.

**Figure 1:**
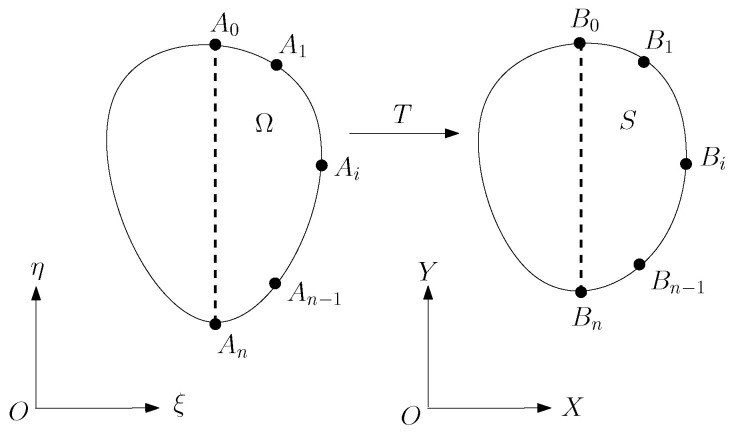
Face transformation from a source face image to a target one.

**Figure 2:**
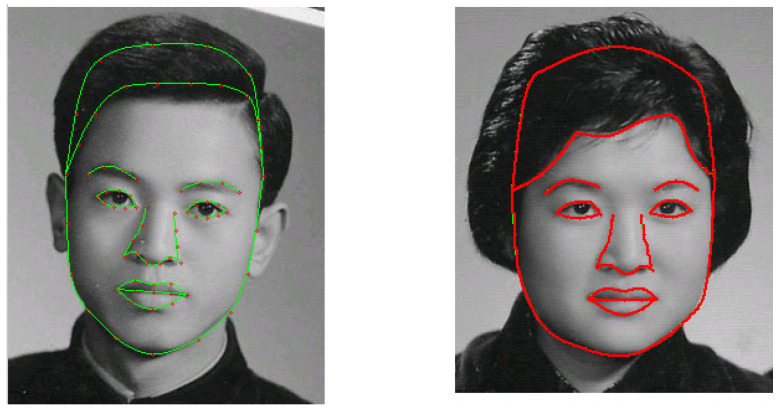
The corresponding exterior and boundary curves of face images of the male (man) and female (woman), where the left shows the blending curves with characteristic nodes, and the right shows the blending curves (with the permission from Z.C. Li).

In (c), the characteristic nodes on the face are related not only to the geometrical properties of facial features, such as the eyes, eyebrows, nose, mouth, etc., but also to the muscle structure of the human face. The significant characteristic nodes are provided in [22]. Those nodes may be found using skeleton techniques and by locating the boundary nodes, the sharp nodes of curves, and the nodes of inflection. Since the number of pixels contained in an image boundary is large, it is laborious to manually establish pixel correspondence between two boundaries to be matched, as shown in Figure 1 and Figure 2. New methods are solicited to formulate and map the two boundary curves only by a few matching characteristic points given.

Suppose that the given (n+1) matching pairs of characteristic points $A_{i}$ and $B_{i}$ satisfy

$$\begin{array}{ccc} & & A_{i}\overset{T}{\to }B_{i},\;i=0,1,\ldots ,n, \end{array}$$

where $A_{i}\in \partial \Omega$, and $B_{i}\in \partial S$. In Section 2, we will describe the numerical methods used to generate ∂Ω, ∂S, and their mapping in (1). We will use two methods: (M1) the cubic spline in [21] and (M2) ordinary differential equations (ODE) using Hermite interpolation by following [17]. Method (M2) is more flexible and advanced because it can be applied in parametric forms and is well suited to complicated curves, such as the contours of the head, eyes, eyebrows, nose, and ears. The new techniques of ODE can handle complicated boundary conditions for given slopes and curvatures and allow cubic splines to be embedded in the blending curves.

**Figure 3:**
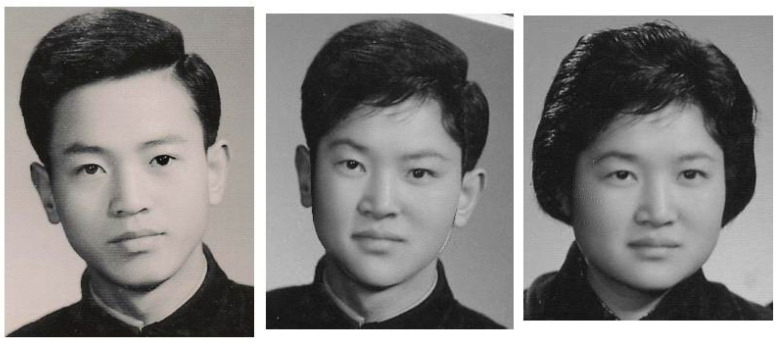
The face transformation from female to male, where the center one is the fusion image (with the permission from Z.C. Li).

In [12], the boundary curves of face images of a male (man) and a female (woman) are formulated, as shown in Figure 2, and the face transformation from the female to the male in shape is given in the center of Figure 3. Face boundary formulation and mapping are completed based on the techniques described in this paper.

Step II: Carry out the harmonic model.

Since the image pixels are distributed uniformly, we may simply choose pixel points as the different nodes $\left(i,j\right)=\left(\xi_{i},\eta_{j}\right)=\left(iH,jH\right)$, where *H* is the pixel size. Denote $x_{i,j}=x\left(i,j\right)$ and $y_{i,j}=y\left(i,j\right)$. Take the Laplace equation Δx=0 in (3) as an example. The simple interior difference equations are given by the following:

(5) $$\begin{array}{ccc} & & x_{i+1,j}+x_{i-1,j}+x_{i,j+1}+x_{i,j-1}-4x_{i,j}=0,\;\left(i,j\right)\in \Omega , \end{array}$$

(6) $$\begin{array}{ccc} & & x\left(i,j\right)={\bar{x}}_{ij},\;\left(i,j\right)\in \partial \Omega , \end{array}$$

where ${\bar{x}}_{ij}$ is known. Note that the number of (5) is nearly that of the total pixels of the original image used to solve the above difference equations. The successive over-relaxation iteration (SOR) in [23] can be used to solve (5) and (6):

$$\begin{array}{ccc} & & x_{ij}^{\left(k+1\right)}=x_{ij}^{\left(k\right)}-\frac{w_{opt}}{4}\left\{4x_{ij}^{\left(k\right)}-\left(x_{i-1,j}^{\left(k+1\right)}+x_{i,j-1}^{\left(k+1\right)}+x_{i+1,j}^{\left(k\right)}+x_{i,j+1}^{\left(k\right)}\right)\right\}, \end{array}$$

where $w_{opt}$ is the optimal relaxation parameter, and $x_{ij}^{\left(0\right)}$ are the given initial values. For the arbitrary face domain Ω, although the optimal parameter $w_{opt}$ in [23] is unknown, some new numerical techniques in Chen [24] are provided to seek the optimal $w_{opt}$ easily by trial computation.

To reduce computation complexity, we may choose some pixels $\left(\bar{i},\bar{j}\right)$ as the difference nodes and establish the non-uniform difference equations. We define $\left(i,j\right)=\left(\xi_{i},\eta_{j}\right)$, $x_{ij}=x\left(\xi_{i},\eta_{j}\right)$, where $\xi_{i}=\bar{i}H$ and $\eta_{j}=\bar{j}H$ are not uniform. The mesh spacings are denoted by $h_{i}=\xi_{i+1}-\xi_{i}$ and $k_{j}=\eta_{j+1}-\eta_{j}$. Hence, for $\frac{\partial^{2}x}{\partial \xi^{2}}+\frac{\partial^{2}x}{\partial \eta^{2}}=f$ in Poisson’s model, the interior difference equations are obtained by direct difference approximations (see Li [25]):

(7) $$\begin{array}{ccc} & & \;\frac{2}{h_{i}+h_{i-1}}\left(\frac{x_{i+1,j}-x_{i,j}}{h_{i}}-\frac{x_{i,j}-x_{i-1,j}}{h_{i-1}}\right)+\frac{2}{k_{j}+k_{j-1}}\left(\frac{x_{i,j+1}-x_{i,j}}{k_{j}}-\frac{x_{i,j}-x_{i,j-1}}{k_{j-1}}\right)=f_{ij}. \end{array}$$

We multiply two sides of (7) by the factor $-\left(\frac{h_{i}+h_{i-1}}{2}\right)\left(\frac{k_{j}+k_{j-1}}{2}\right)$ to yield the symmetric difference equations:

$$\begin{array}{ccc} & & -\left(\frac{k_{j}+k_{j-1}}{2}\right)\left(\frac{x_{i+1,j}-x_{i,j}}{h_{i}}-\frac{x_{i,j}-x_{i-1,j}}{h_{i-1}}\right)-\left(\frac{h_{i}+h_{i-1}}{2}\right)\left(\frac{x_{i,j+1}-x_{i,j}}{k_{j}}-\frac{x_{i,j}-x_{i,j-1}}{k_{j-1}}\right)\\ & & =-\left(\frac{h_{i}+h_{i-1}}{2}\right)\left(\frac{k_{j}+k_{j-1}}{2}\right)f_{ij},\;\left(i,j\right)\in S. \end{array}$$

FDM is simpler than FEM and FVM, and its programming is easy. As a result, FDM is beneficial to the images of medium size converted by harmonic transformations. For example, in Section 4, with near 500×500 pixels, the computation of FDM by the SOR with the optimal parameter $w_{opt}$ can be computed very quickly by using a personal computer.

**Figure 4:**
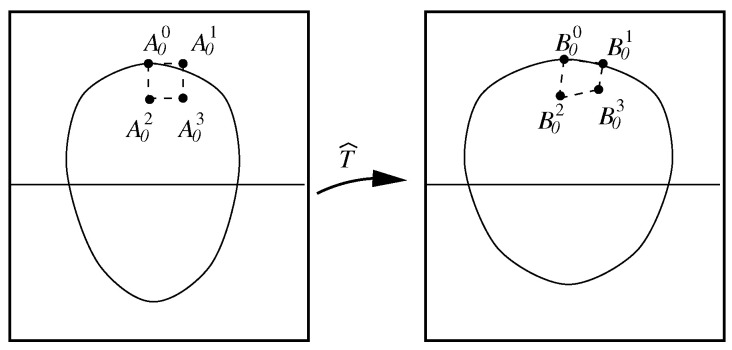
Piecewise bilinear transformations.

**Figure 5:**
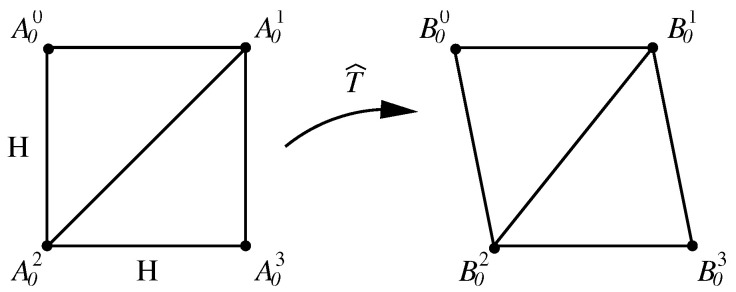
Piecewise bilinear transformations on a square.

After the solutions $\left(x_{i,j},y_{i,j}\right)$ have been obtained from FDM, the piecewise bilinear transformations are given by $x=\hat{x}\left(\xi ,\eta \right)$ and $y=\hat{y}\left(\xi ,\eta \right)$. The functions $\left(\hat{x}\left(\xi ,\eta \right),\hat{y}\left(\xi ,\eta \right)\right)$ are approximated to (x(ξ,η),y(ξ,η)) of the harmonic transformation with $A_{0}^{i}\overset{T}{\to }B_{0}^{i},i=0,1,2,3$. The functions $\hat{x}$ and $\hat{y}$ inside are obtained by the bilinear interpolations in Figure 4 or simply by the linear interpolations in Figure 5. Note that $A_{0}^{i}$ should be located at the pixel points in ξOη, but it is not necessary for $B_{0}^{i}$ in XOY.

Step III: Use combinations of the SIM in Li [13] to obtain the distorted and restored images.

It is worth pointing out that the numerical algorithms via Steps I–III in this paper are beneficial not only to face transformations but also to image geometric transformations. We cite the following related reports: Castleman [26], Lakemond et al. [27], Rosa et al. [28], Luhmann et al. [29], Holden [30], Li et al. [31], Ma et al. [32], Gao et al. [33], Fang et al. [34], Chen et al. [35], Tian et al. [36], You et al. [37], and Püspöki [38].

This paper is organized as follows: in Section 2, the blending techniques and their applications are explored for Step I. In Section 3, the numerical methods for Steps II and III are addressed. In Section 4, some image examples of face transformations and resembling are provided. In Section 5, a few concluding remarks are addressed. In Appendix A, a proof of Theorem 1 is presented, and in Appendix B, the combinations of cubic splines and ODE approaches are discussed.

## 2. Curves for Face Boundaries

**Figure 6:**
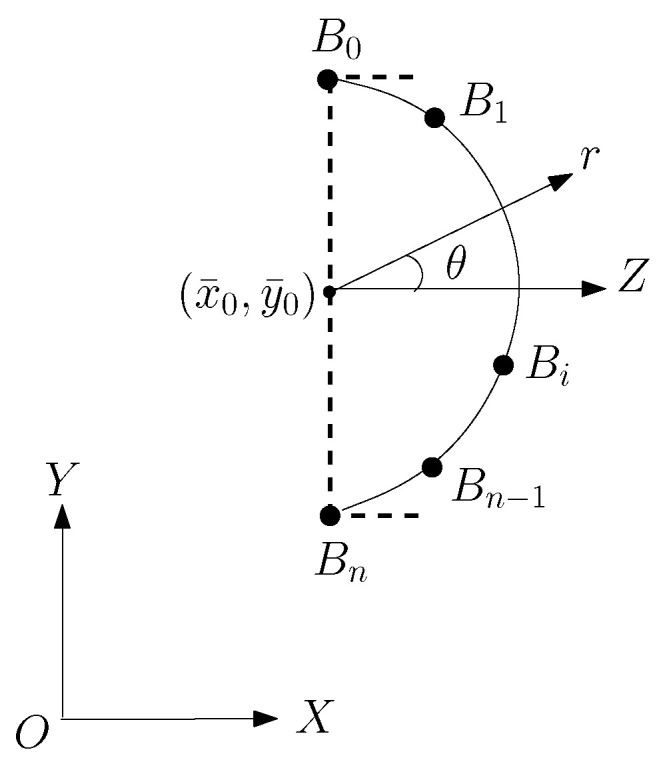
Right half of face contour.

For simplicity, we consider the right boundary of a face in XOY in Figure 6. We choose the nose’s central point as the origin of polar coordinates, where $\vec{z}$ is parallel to direction $\vec{x}$. The right-half boundary ∂S of the half-face contour can be denoted by

$$\begin{array}{ccc} & & r=r\left(\theta \right),\;-\frac{\pi }{2}\leq \theta \leq \frac{\pi }{2}. \end{array}$$

We assume the ending conditions of ∂S are just horizontal,

(8) $$\begin{array}{ccc} & & r^{\prime }\left(-\frac{\pi }{2}\right)=r^{\prime }\left(\frac{\pi }{2}\right)=0. \end{array}$$

Equation (8) makes the curve figuration easier to connect the right half-face contour, see Figure 1.

Suppose that $B_{i}$ is located in $\left(x_{i},y_{i}\right)$ in XOY, where $x_{i}=k_{i}H$, and $y_{i}=j_{i}H$. The origin of the (Cartesian) coordinates is located at $\left({\bar{x}}_{0},{\bar{y}}_{0}\right)$, with ${\bar{x}}_{0}=\bar{i}H$ and ${\bar{y}}_{0}=\bar{j}H$, where *H* is the mesh resolution. Hence, the polar coordinates of point $B_{i}=\left(r,\theta \right)$ are given by

$$\begin{array}{ccc} & & r=r\left(B_{i}\right)=\sqrt{{\left(x_{i}-{\bar{x}}_{0}\right)}^{2}+{\left(y_{i}-{\bar{y}}_{0}\right)}^{2}}=\sqrt{{\left(k_{i}-\bar{i}\right)}^{2}+{\left(j_{i}-\bar{j}\right)}^{2}}H,\\ & & \theta =\theta \left(B_{i}\right)=\arctan\frac{y_{i}-\bar{y}}{x_{i}-\bar{x}}=\arctan\frac{j_{i}-\bar{j}}{k_{i}-\bar{i}}. \end{array}$$

For simplicity, we only choose the beginning and end points located at the vertical line: $x\left(B_{0}\right)=x\left(B_{n}\right)={\bar{x}}_{0}$, see Figure 6. Two interpolation methods are provided below. The cubic splines are well-known, and the ODE using the Hermite interpolation for blending curves is new and developed from [17].

### 2.1. Cubic Spline Interpolation

**Figure 7:**
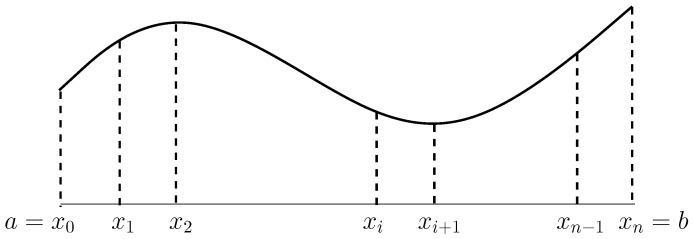
Cubic spline interpolation.

Consider the piecewise cubic polynomials $s\left(x\right)\in C^{2}\left[a,b\right]$, where $C^{k}\left[a,b\right]$ denotes the set of functions having *k*-order continuous derivatives. Choose $a=x_{0}<x_{1}<\ldots <x_{n}=b$ and $h_{i}=x_{i}-x_{i-1}$. Assume that $(x_{i},y_{i}),i=0,1,\ldots ,n$ are given and the derivatives $y_{0}^{\prime }$ and $y_{n}^{\prime }$ on two boundary nodes are known (e.g., $y_{0}^{\prime }=y_{n}^{\prime }=0$ in Figure 7). For the function s(x), denote

$$\begin{array}{ccc} & & s\left(x_{i}\right)=y_{i},\;\;s^{\prime }\left(x_{i}\right)=m_{i},\;\;s^{\prime \prime }\left(x_{i}\right)=M_{i},\;\;i=0,1,2,\ldots ,n. \end{array}$$

Then, the piecewise cubic polynomials s(x) are given by (see Su and Liu [21] p. 13)

(9) $$\begin{array}{ccc} & & s\left(x\right)=\frac{M_{i-1}}{6h_{i}}{\left(x_{i}-x\right)}^{3}+\frac{M_{i}}{6h_{i}}{\left(x-x_{i-1}\right)}^{3}+\left(\frac{y_{i-1}}{h_{i}}-\frac{h_{i}M_{i-1}}{6}\right)\left(x_{i}-x\right)\\ & & +\left(\frac{y_{i}}{h_{i}}-\frac{h_{i}M_{i}}{6}\right)\left(x-x_{i-1}\right),\;\mathrm{in}\;\left[x_{i-1},x_{i}\right]. \end{array}$$

For the given boundary derivatives $m_{0}$ and $m_{n}$, the values of $m_{i},i=1,\ldots ,n-1$ can be obtained from the linear algebraic system,

(10) $$\begin{array}{ccc} & & \lambda_{i}m_{i-1}+2m_{i}+\mu_{i}m_{i+1}=C_{i},\;\;i=1,2,\ldots ,n-1, \end{array}$$

where the constants are

$$\begin{array}{ccc} & & \lambda_{i}=\frac{h_{i+1}}{h_{i}+h_{i+1}},\;\mu_{i}=\frac{h_{i}}{h_{i}+h_{i+1}},\;C_{i}=3\left(\lambda_{i}\frac{y_{i}-y_{i-1}}{h_{i}}+\mu_{i}\frac{y_{i+1}-y_{i}}{h_{i+1}}\right). \end{array}$$

Equations (10) can be easily solved due to the tri-diagonal coefficient matrix. Then, the values $M_{i},i=1,\ldots ,n-1$ of second-order derivatives can be found by

$$\begin{array}{ccc} & & M_{i}=s^{\prime \prime }\left(x_{i}^{-}\right)=\frac{2m_{i-1}}{h_{i}}+\frac{4m_{i}}{h_{i}}-6\frac{y_{i}-y_{i-1}}{h_{i}^{2}}, \end{array}$$

or

$$\begin{array}{ccc} & & M_{i}=s^{\prime \prime }\left(x_{i}^{+}\right)=-\frac{4m_{i}}{h_{i+1}}-\frac{2m_{i+1}}{h_{i+1}}+6\frac{y_{i+1}-y_{i}}{h_{i+1}^{2}}, \end{array}$$

where $x_{i}^{-}$ and $x_{i}^{+}$ are the one-side limit of $x_{i}$ from the left and right, respectively. Once $m_{i}$ and $M_{i}$ are given, the entire function s(x) in (9) is provided.

### 2.2. Ordinary Differential Equations Using the Hermite Interpolation

**Figure 8:**
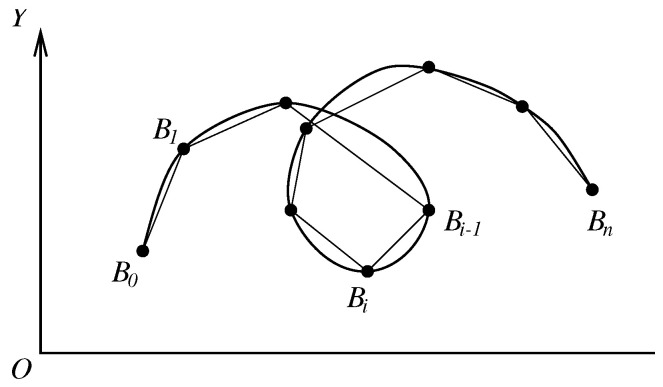
General 2D curves.

For general 2D curves, since suitable Cartesian coordinates (or polar coordinates) cannot be found, the parametric functions are chosen instead (see Figure 8).

$$\begin{array}{ccc} & & x=x(S),\;y=y(S),\;0\leq S\leq 1. \end{array}$$

In [21] (p. 69), the cumulative chord length can be chosen as the following variables:

$$\begin{array}{ccc} & & t_{i}=|{\bar{B_{i}B}}_{i-1}|=\sqrt{{\left(x_{i}-x_{i-1}\right)}^{2}+{\left(y_{i}-y_{i-1}\right)}^{2}},\;1\leq i\leq n. \end{array}$$

Define

$$\begin{array}{ccc} & & S_{i}=\frac{\sum_{j=1}^{i}t_{j}}{\sum_{j=1}^{n}t_{j}}; \end{array}$$

then, $0\leq S_{i}\leq 1$. We follow [17] to form the Hermite functions satisfying ODE. Consider x(S) and y(S) on [0,1] with the nodes $S_{i}$, $0=S_{0}<S_{1}<\ldots <S_{n}=1$. On $\left[S_{i},S_{i+1}\right]$, we choose the Hermite interpolations

(11) $$\begin{array}{ccc} & & \;x_{h}^{i}\left(S\right)=x_{i}\phi_{0}\left(\frac{S-S_{i}}{h_{i+1}}\right)+x_{i+1}\phi_{1}\left(\frac{S-S_{i}}{h_{i+1}}\right)+h_{i+1}\left[x_{i}^{\prime }\psi_{0}\left(\frac{S-S_{i}}{h_{i+1}}\right)+x_{i+1}^{\prime }\psi_{1}\left(\frac{S-S_{i}}{h_{i+1}}\right)\right], \end{array}$$

where $h_{i+1}=S_{i+1}-S_{i}$, $x_{i}=x\left(S_{i}\right)$, and $x_{i}^{\prime }=x^{\prime }\left(S_{i}\right)$. The Hermite basis functions are

(12) $$\begin{array}{ccc} & & \;\phi_{0}\left(\bar{S}\right)=2{\bar{S}}^{3}-3{\bar{S}}^{2}+1,\;\phi_{1}\left(\bar{S}\right)=-2{\bar{S}}^{3}+3{\bar{S}}^{2},\;\psi_{0}\left(\bar{S}\right)={\bar{S}}^{3}-2{\bar{S}}^{2}+\bar{S},\;\psi_{1}\left(\bar{S}\right)={\bar{S}}^{3}-{\bar{S}}^{2}, \end{array}$$

where $\bar{S}\in \left[0,1\right]$. Assume that

$$\begin{array}{ccc} & & {\left.\frac{dy}{dx}\right|}_{S=0,1}=\frac{y^{\prime }\left(S\right)}{x^{\prime }\left(S\right)}=0. \end{array}$$

Then, we have the following boundary conditions:

(13) $$\begin{array}{ccc} & & \left(1\right)\;x_{0},x_{n},y_{0},\;\mathrm{and}\;y_{n}\;\mathrm{are}\;\mathrm{given},\;\;y_{0}^{\prime }=y^{\prime }\left(0\right)=0,\;\;y_{n}^{\prime }=y^{\prime }\left(1\right)=0. \end{array}$$

(14) $$\begin{array}{ccc} & & \left(2\right)\;x_{0}^{\prime }\;\mathrm{and}\;x_{n}^{\prime }\;\mathrm{arbitrary}. \end{array}$$

Equations (13) and (14) enable us to separate the ODE.

The blending curves are modeled as follows. Consider the single equation of the fourth-order ODE

$$\begin{array}{ccc} & & \frac{d^{2}}{dS^{2}}\left(p^{x}\left(S\right)\frac{d^{2}}{dS^{2}}x\left(S\right)\right)=f^{x}\left(S\right),\;0\leq S\leq 1, \end{array}$$

where $p^{x}\left(S\right)\left(>0\right)$ is the reflection coefficient of the material, and $f^{x}\left(S\right)$ denotes the outward force. The boundary conditions $x_{0}$ and $x_{n}$ are given, but $x_{0}^{\prime }$ and $x_{n}^{\prime }$ may be zero or arbitrary. Hence, the variational description is employed. Denote $V_{h}$ as the space of the piecewise Hermite functions (11) satisfying the given boundary conditions. Also, define the energy as follows:

$$\begin{array}{ccc} & & I_{x}\left(x\left(S\right)\right)=\frac{1}{2}\int_{0}^{1}p^{x}\left(S\right){\left(\frac{d^{2}x}{dS^{2}}\right)}^{2}\;dS-\int_{0}^{1}f^{x}\left(S\right)x\left(S\right)\;dS. \end{array}$$

We seek the minimum solution $x_{h}$ by

(15) $$\begin{array}{ccc} & & I_{x}\left(x_{h}\left(S\right)\right)=\min_{\forall x\left(S\right)\in V_{h}}I_{x}\left(x\left(S\right)\right). \end{array}$$

For conditions (13) and (14), $x_{i}$ and $y_{i}$ are given, but $x_{i}^{\prime }\;\left(i=0,1,\ldots ,n\right)$ and $y_{i}^{\prime }\;\left(i=1,\ldots ,n-1\right)$ are unknown, for which the difference equations will be established. Take the difference equations for $x_{i}^{\prime }$, for example, since the equations for $y_{i}^{\prime }$ are similar. The equations for $x_{i}^{\prime }$ can be obtained from (15) (see [17]) and given as follows. We choose Simpson’s rule to approximate the integration and denote $\tilde{I_{x}}\;\left(\approx I_{x}\right)$. The interior difference equations for i=1,2,…,n−1 are given by the following:

(16) $$\begin{array}{ccc} & & 0=\frac{\partial }{\partial x_{i}^{\prime }}\tilde{I_{x}}\left(\vec{X}\right)={\bar{a}}_{i}^{x}x_{i-1}+{\bar{b}}_{i}^{x}x_{i}+{\bar{c}}_{i}^{x}x_{i+1}+{\bar{d}}_{i}^{x}x_{i-1}^{\prime }+{\bar{e}}_{i}^{x}x_{i}^{\prime }+{\bar{g}}_{i}^{x}x_{i+1}^{\prime }+{\bar{r}}_{i}^{x}, \end{array}$$

where the constants are as follows:

$$\begin{array}{ccc} & & {\bar{a}}_{i}^{x}=\frac{2}{h_{i}^{2}}\left(2p_{i}^{x}+p_{i-1}^{x}\right),\;\;{\bar{b}}_{i}^{x}=-\left({\bar{a}}_{i}^{x}+{\bar{c}}_{i}^{x}\right),\\ & & {\bar{c}}_{i}^{x}=-\frac{2}{h_{i+1}^{2}}\left(p_{i+1}^{x}+2p_{i}^{x}\right),\;\;{\bar{d}}_{i}^{x}=\frac{4}{3}\frac{1}{h_{i}}\left(p_{i}^{x}+p_{i-1}^{x}\right)-\frac{2}{3}\frac{p_{i-\frac{1}{2}}^{x}}{h_{i}},\\ & & {\bar{e}}_{i}^{x}=\frac{1}{3h_{i+1}}\left(2p_{i+1}^{x}+8p_{i}^{x}\right)+\frac{1}{3h_{i}}\left(8p_{i}^{x}+2p_{i-1}^{x}\right)+\frac{2}{3}\left(\frac{p_{i+\frac{1}{2}}^{x}}{h_{i+1}}+\frac{p_{i-\frac{1}{2}}^{x}}{h_{i}}\right),\\ & & {\bar{g}}_{i}^{x}=\frac{4}{3}\frac{1}{h_{i+1}}\left(p_{i+1}^{x}+p_{i}^{x}\right)-\frac{2}{3}\frac{p_{i+\frac{1}{2}}^{x}}{h_{i+1}},\;\;{\bar{r}}_{i}^{x}=-\frac{1}{12}(f_{i+\frac{1}{2}}^{x}h_{i+1}^{2}-f_{i-\frac{1}{2}}^{x}h_{i}^{2})\;. \end{array}$$

Next, the boundary difference equation for $x_{0}^{\prime }$ is given by

(17) $$\begin{array}{ccc} & & 0=\frac{\partial }{\partial x_{0}^{\prime }}\tilde{I_{x}}\left(\vec{X}\right)={\bar{b}}_{0}^{x}x_{0}+{\bar{c}}_{0}^{x}x_{1}+{\bar{e}}_{0}^{x}x_{0}^{\prime }+{\bar{g}}_{0}^{x}x_{1}^{\prime }+{\bar{r}}_{0}^{x}, \end{array}$$

where the constants are as follows:

$$\begin{array}{ccc} {\bar{b}}_{0}^{x} & = & \frac{1}{h_{1}^{2}}\left(4p_{0}^{x}+2p_{1}^{x}\right),\;\;{\bar{c}}_{0}^{x}=-{\bar{b}}_{0}^{x},\;{\bar{e}}_{0}^{x}=\frac{2}{3h_{1}}(4p_{0}^{x}+p_{1}^{x}+p_{\frac{1}{2}}^{x}),\\ {\bar{g}}_{0}^{x} & = & \frac{2}{3h_{1}}(2p_{0}^{x}+2p_{1}^{x}-p_{\frac{1}{2}}^{x}),\;\;{\bar{r}}_{0}^{x}=-\frac{1}{12}f_{\frac{1}{2}}^{x}h_{1}^{2}\;. \end{array}$$

Last, the boundary difference equation for $x_{n}^{\prime }$ is given by

(18) $$\begin{array}{ccc} & & 0=\frac{\partial }{\partial x_{n}^{\prime }}\tilde{I_{x}}\left(\vec{X}\right)={\bar{a}}_{n}^{x}x_{n-1}+{\bar{b}}_{n}^{x}x_{n}+{\bar{d}}_{n}^{x}x_{n-1}^{\prime }+{\bar{e}}_{n}^{x}x_{n}^{\prime }+{\bar{r}}_{n}^{x}, \end{array}$$

where the constants are as follows:

$$\begin{array}{ccc} & & {\bar{a}}_{n}^{x}=\frac{1}{h_{n}^{2}}\left(2p_{n-1}^{x}+4p_{n}^{x}\right),\;\;{\bar{b}}_{n}^{x}=-{\bar{a}}_{n}^{x},\;\;{\bar{d}}_{n}^{x}=\frac{2}{3h_{n}}(2p_{n-1}^{x}+2p_{n}^{x}-p_{n-\frac{1}{2}}^{x}),\\ & & {\bar{e}}_{n}^{x}=\frac{2}{3h_{n}}(p_{n-1}^{x}+4p_{n}^{x}+p_{n-\frac{1}{2}}^{x}),\;\;{\bar{r}}_{n}^{x}=\frac{1}{12}h_{n}^{2}f_{n-\frac{1}{2}}^{x}. \end{array}$$

**Figure 9:**
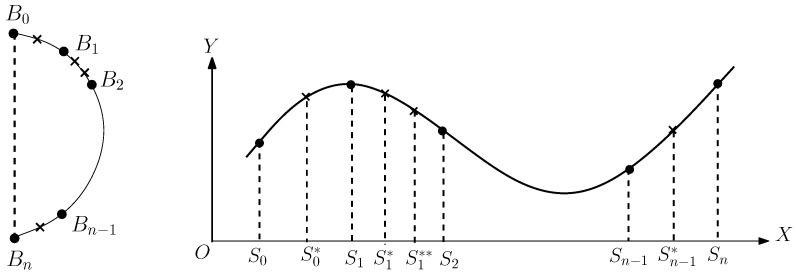
Adding intermediate points.

**Figure 10:**
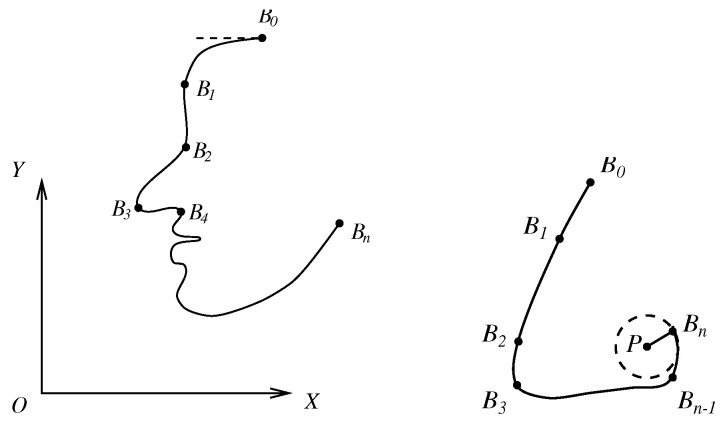
A face contour (**left**) and nose contour (**right**).

For the right half boundary, we may use the ODE techniques as in Figure 9 to seek more intermediate points. Once $x_{i}$ and $x_{i}^{\prime }$ are obtained, we find the intermediate points from the Hermite functions in (11). Note that the curves of the Hermite functions by ODE have minimal energy, which grants the curves good smooth properties. For the given slope and curvature at the curve boundary in Figure 10, the numerical techniques are provided by Chen [24].

We provide the following theorem, whose proofs are given in Appendix A.

**Theorem** **1.** *Denote the energy*

(19) $$\begin{array}{ccc} & & E^{*}\left(y\left(S\right)\right)=\frac{1}{2}\int_{0}^{1}p\left(S\right){\left(\frac{d^{2}y}{dS^{2}}\right)}^{2}\;dS-\int_{0}^{1}f\left(S\right)y\left(S\right)\;dS. \end{array}$$

Let $y_{h}\left(S\right)$ be the Hermite functions given by $E^{*}\left(y_{h}\left(S\right)\right)=\min_{\forall y\left(S\right)\in V_{h}}E^{*}\left(y\left(S\right)\right)$ with p(S)≡1 and f≡0. Then, the minimal solution of $E^{*}\left(y\right)$ gives the exact cubic spline functions satisfying $y\in C^{2}\left[0,1\right]$ and (13).

Furthermore, the new combinations of cubic splines and ODE approaches are explored in Appendix B.

### 2.3. Intermediate Pixel–Pixel Correspondence Between Two Face Boundaries

**Figure 11:**
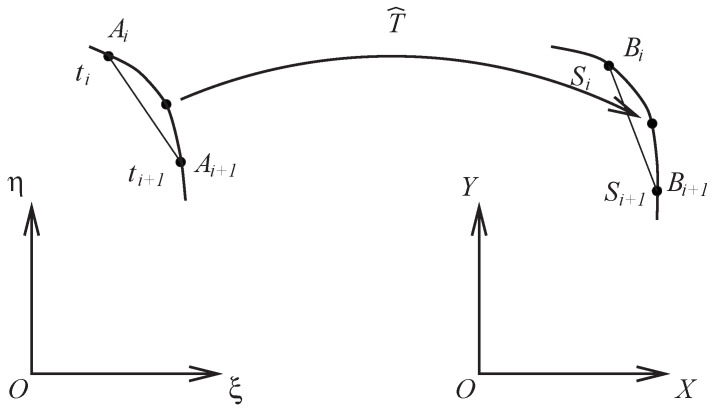
Intermediate points.

To map two face boundaries (1), the intermediate points between $A_{i}$ and $A_{i+1}$ and $B_{i}$ and $B_{i+1}$ can also be used (see Figure 11):

$$\begin{array}{ccc} & & \xi =\xi \left(t\right),\;\;\eta =\eta \left(t\right),\;\;t_{i}<t_{i+1},\;\;A_{i}\to A_{i+1},\\ & & x=x\left(S\right),\;\;y=y\left(S\right),\;\;S_{i}<S_{i+1},\;\;B_{i}\to B_{i+1}. \end{array}$$

Consider the function S=S(t), $0<t_{i}<t_{i+1}<1,0<S_{i}<S_{i+1}<1$. The simplest function of S(t) is the piecewise linear interpolation:

$$\begin{array}{ccc} & & S=S_{i}+\frac{S_{i+1}-S_{i}}{t_{i+1}-t_{i}}\left(t-t_{i}\right). \end{array}$$

However, the spline cubic interpolation and the ODE using the Hermite interpolation provide better intermediate pixels.

## 3. Splitting–Integrating Method for the Image Grayness of Harmonic Transformations

The splitting–integrating method (SIM) originated in [14] and was then developed in both algorithms and error analysis in [13]. The image grayness $\Phi_{ij}$ and $B_{IJ}$ can be represented by the mean of continuous (or piecewise continuous) intensity functions ϕ(ξ,η) and b(x,y):

(20) $$\begin{array}{ccc} & & \Phi_{ij}=\frac{1}{H^{2}}\int \;\;\;\int_{{□}_{ij}}\phi \left(\xi ,\eta \right)\;d\xi d\eta ,\;\;B_{IJ}=\frac{1}{H^{2}}\int \;\;\;\int_{{□}_{IJ}}b\left(x,y\right)\;dxdy,\;\;\; \end{array}$$

where b(x,y)=ϕ(ξ(x,y),η(x,y)), and

(21) $$\begin{array}{ccc} & & {□}_{ij}=\left\{\left(\xi ,\eta \right)|\left(i-\frac{1}{2}\right)H\leq \xi <\left(i+\frac{1}{2}\right)H,\left(j-\frac{1}{2}\right)H\leq \eta <\left(j+\frac{1}{2}\right)H\right\}, \end{array}$$

(22) $$\begin{array}{ccc} & & {□}_{IJ}=\left\{\left(x,y\right)|\left(I-\frac{1}{2}\right)H\leq x<\left(I+\frac{1}{2}\right)H,\left(J-\frac{1}{2}\right)H\leq y<\left(J+\frac{1}{2}\right)H\right\}. \end{array}$$

Consider an image with 256 gray levels. Numerical methods are used to evaluate image grayness under harmonic transformation. In [12], the splitting–shooting method (SSM) and the splitting–integrating method (SIM) are used for harmonic transformation *T* and its inversion transformation $T^{-1}$, respectively. In this paper, we choose the SIM for both *T* and $T^{-1}$. The SIM is well suited to harmonic transformation since the sequential convergence of $O(N^{-2})$ is higher than $O(N^{-1.5})$ in the SSM (see [13]).

### 3.1. Splitting–Integrating Method (SIM) for $T^{-1}$

First, consider the inverse transformation $T^{-1}:\left(x,y\right)⟶\left(\xi ,\eta \right)$. From the known distorted image pixels $\left\{{\hat{Z}}_{IJ}\right\}$, the approximate functions

$$\begin{array}{ccc} & & b\left(x,y\right)\approx b_{\mu }\left(x,y\right)=\phi_{\mu }\left(\xi \left(x,y\right),\eta \left(x,y\right)\right), \end{array}$$

where μ is the order of grayness interpolations, and μ=0 and μ=1 denote the constant and the linear interpolation, respectively (see [14]). Below, the composite centroid rule will be used to evaluate the integration values in (20). Let ${□}_{ij}$ in (21) be split into N×N uniform squares ${□}_{ij,k\ell }$, i.e., ${□}_{ij}={⋃}_{k,\ell }{□}_{ij,k\ell }$, where

$$\begin{array}{c} {□}_{ij,k\ell }=\left\{\begin{array}{cc} (\xi ,\eta ), & \left(i-\frac{1}{2}\right)H+\left(k-1\right)h\leq \xi <\left(i-\frac{1}{2}\right)H+kh,\\ & \left(j-\frac{1}{2}\right)H+\left(\ell -1\right)h\leq \eta <\left(j-\frac{1}{2}\right)H+\ell h \end{array}\right\}, \end{array}$$

where *h* is the boundary length of ${□}_{ij,k\ell }$ given by $h=\frac{H}{N}$. For small subpixels ${□}_{ij,k\ell }$, the coordinates of the center of gravity are given by

$$\begin{array}{ccc} & & \xi_{\dot{G}}=\xi_{{\dot{G}}_{ij,k\ell }}=\left(i-\frac{1}{2}\right)H+\left(k-\frac{1}{2}\right)h,\;\;\eta_{\dot{G}}=\eta_{{\dot{G}}_{ij,k\ell }}=\left(j-\frac{1}{2}\right)H+\left(\ell -\frac{1}{2}\right)h. \end{array}$$

Based on the composite centroid rule, we have

(23) $$\begin{array}{ccc} & & \int \;\;\;\int_{{□}_{ij,k\ell }}\phi \left(\xi ,\eta \right)\;d\xi d\eta =\int \;\;\;\int_{{□}_{ij,k\ell }}b\left(x\left(\xi ,\eta \right),y\left(\xi ,\eta \right)\right)\;d\xi d\eta \\ & & \approx h^{2}b\left(x\left(\xi_{{\dot{G}}_{ij,k\ell }},\eta_{{\dot{G}}_{ij,k\ell }}\right),y\left(\xi_{{\dot{G}}_{ij,k\ell }},\eta_{{\dot{G}}_{ij,k\ell }}\right)\right). \end{array}$$

Hence, the normalized image grayness of ${\hat{W}}_{ij}$ at pixel (i,j) is obtained by

$$\begin{array}{ccc} & & \Phi_{ij}\approx \frac{h^{2}}{H^{2}}\sum_{k,\ell =1}^{N}b_{\mu }\left(x\left(\xi_{{\dot{G}}_{ij,k\ell }},\eta_{{\dot{G}}_{ij,k\ell }}\right),y\left(\xi_{{\dot{G}}_{ij,k\ell }},\eta_{{\dot{G}}_{ij,k\ell }}\right)\right). \end{array}$$

Note that the computational algorithms (23) do not involve any nonlinear solutions, and the sequential errors as μ=1 are proven to be $O(N^{-2})$ in [13].

### 3.2. Harmonic Face Image Transformation *T*

Let ${□}_{IJ}$ in (22) be split into N×N uniform squares, i.e., ${□}_{IJ}={⋃}_{k,\ell }{□}_{IJ,k\ell }$. The pixel grayness via *T* is also evaluated by the composite centroid rule,

(24) $$\begin{array}{ccc} B_{IJ} & = & \frac{1}{H^{2}}\sum_{k,\ell =1}^{N}\int \;\;\;\int_{{□}_{IJ,k\ell }}b\left(x,y\right)\;dxdy\\ & \approx & \frac{1}{H^{2}}\sum_{k,\ell =1}^{N}\int \;\;\;\int_{{□}_{IJ,k\ell }}\phi_{\mu }\left(\xi \left(x,y\right),\eta \left(x,y\right)\right)\;dxdy\\ & \approx & (\frac{h}{H}{)}^{2}\sum_{k,\ell =1}^{N}\phi_{\mu }\left(\xi \left(x_{\dot{g}},y_{\dot{g}}\right),\eta \left(x_{\dot{g}},y_{\dot{g}}\right)\right):=B_{IJ}^{\left(N\right)}, \end{array}$$

where $\left(x_{\dot{g}},y_{\dot{g}}\right)$ is the gravity center of ${□}_{IJ,k\ell }$. In (24), we do need the values $\xi_{\dot{g}}=\xi \left(x_{\dot{g}},y_{\dot{g}}\right)$ and $\eta_{\dot{g}}=\eta \left(x_{\dot{g}},y_{\dot{g}}\right)$, where $x_{\dot{g}}=x\left(\xi_{\dot{g}},\eta_{\dot{g}}\right)$ and $y_{\dot{g}}=y\left(\xi_{\dot{g}},\eta_{\dot{g}}\right)$. In general, to find $\left(\xi_{\dot{g}},\eta_{\dot{g}}\right)$, we need to solve the two nonlinear equations in (2) for the SIM. However, since the approximate harmonic functions are piecewise linear functions, as in Figure 5, the nonlinear solutions are bypassed. Hence, for the harmonic transformation *T* as μ=1, we may retain the convergence rate $O(N^{-2})$ by the SIM in [13], higher than $O(N^{-1.5})$ by the SSM in [12].

Under the harmonic models, the approximate values ${\hat{x}}_{\dot{g}}$ and ${\hat{y}}_{\dot{g}}$, are obtained from the FDM in $\hat{x}\left(\xi ,\eta \right)\approx x\left(\xi ,\eta \right)$ and $\hat{y}\left(\xi ,\eta \right)\approx y\left(\xi ,\eta \right)$, see Section 1.2. Suppose that the transformation *T* is approximated by the piecewise bilinear transformation $\hat{T}$. Then, when the values $\left({\hat{x}}_{ij},{\hat{y}}_{ij}\right)$($\leftarrow (\xi_{ij},\eta_{ij}$)) are known, the values of $\left(\xi_{\dot{g}},\eta_{\dot{g}}\right)$ can be easily found. In fact, the FDM can be regarded as a special kind of finite element method using piecewise linear and bilinear interpolant functions and using special rules of integration approximations (see [25]). Therefore, the FDM solutions $\left({\hat{x}}_{ij},{\hat{y}}_{ij}\right)$ may formulate the piecewise linear transformation $\hat{T}$:

$$\begin{array}{ccc} & & \left(\xi ,\eta \right)\to \left(\hat{x}\left(\xi ,\eta \right),\hat{y}\left(\xi ,\eta \right)\right),\;\;\;\;\left(\xi_{ij},\eta_{ij}\right)\overset{\hat{T}}{⟶}\left({\hat{x}}_{ij},{\hat{y}}_{ij}\right). \end{array}$$

Based on the piecewise bilinear interpolation functions, $\left(\hat{\xi },\hat{\eta }\right)$ are formulated from the known $\left({\hat{\xi }}_{IJ},{\hat{\eta }}_{IJ}\right)$ by the following:

$$\begin{array}{ccc} & & \xi \left(x,y\right)\approx \hat{\xi }\left(x,y\right)={\hat{\xi }}_{I+1,J+1}\frac{\left(x-IH)(y-JH\right)}{H^{2}}+{\hat{\xi }}_{I,J+1}\frac{\left((I+1)H-x)(y-JH\right)}{H^{2}}\\ & & +{\hat{\xi }}_{I+1,J}\frac{\left(x-IH)(y-(J+1)H\right)}{H^{2}}+{\hat{\xi }}_{I,J}\frac{\left((I+1)H-x)((J+1)H-y\right)}{H^{2}}. \end{array}$$

Then, the redesigned SIM of (24) is given by

(25) $$\begin{array}{ccc} & & B_{IJ}\approx {\hat{B}}_{IJ}^{\left(N\right)}=\frac{h^{2}}{H^{2}}\sum_{k,\ell }{\hat{\phi }}_{\mu }\left(\hat{\xi }\left(x_{\dot{g}},y_{\dot{g}}\right),\hat{\eta }\left(x_{\dot{g}},y_{\dot{g}}\right)\right)=\frac{h^{2}}{H^{2}}\sum_{k,\ell }{\hat{b}}_{\mu }\left(x_{\dot{g}},y_{\dot{g}}\right), \end{array}$$

where $\dot{g}={\dot{g}}_{IJ,k\ell }$ and ${\hat{b}}_{\mu }\left(x,y\right)={\hat{\phi }}_{\mu }\left(\hat{\xi }\left(x,y\right),\hat{\eta }\left(x,y\right)\right)$. Note that no nonlinear solutions are needed in (25) either. Here, only one question remains: How do we find $\left({\hat{\xi }}_{IJ},{\hat{\eta }}_{IJ}\right)$ from the FDM solutions $\left({\hat{x}}_{ij},{\hat{y}}_{ij}\right)$? Now, we propose a new technique consisting of four mini steps.

**Figure 12:**
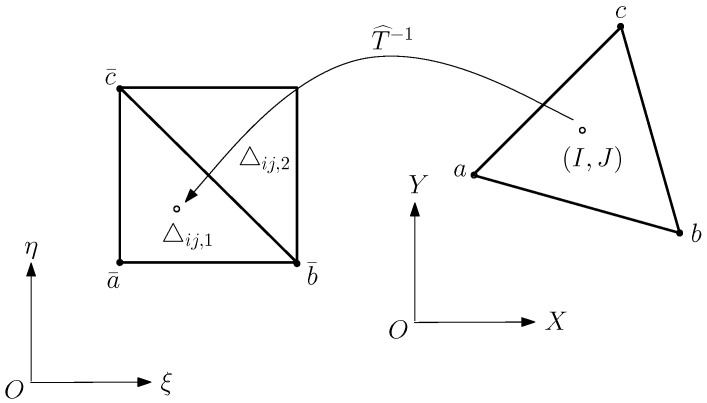
The inverse transformation at (I,J) in XOY.

Step (a): Compute all ${\hat{x}}_{ij}$ and ${\hat{y}}_{ij}$ by the FDM in Step II of Section 2.1,
  $$\begin{array}{ccc} & & {\hat{x}}_{ij}=\hat{x}\left(\xi_{i},\eta_{j}\right)=\hat{x}\left(iH,jH\right),\;\;{\hat{y}}_{ij}=\hat{y}\left(\xi_{i},\eta_{j}\right)=\hat{y}\left(iH,jH\right). \end{array}$$
Step (b): Find potential candidates $\left(I,J\right)\in {\hat{\Omega }}_{IJ}\;\left({\hat{\Omega }}_{ij}\overset{\hat{T}}{⟵}{□}_{ij}\right)$, which may be determined by $I_{\min}\leq I\leq I_{\max}$ and $J_{\min}\leq J\leq J_{\max}$, where
  $$\begin{array}{ccc} & & I_{\max}=\max\left\{\left\lfloor \frac{{\hat{x}}_{ij}}{H}\right\rfloor ,\left\lfloor \frac{{\hat{x}}_{i+1,j}}{H}\right\rfloor ,\left\lfloor \frac{{\hat{x}}_{i,j+1}}{H}\right\rfloor ,\left\lfloor \frac{{\hat{x}}_{i+1,j+1}}{H}\right\rfloor \right\},\\ & & J_{\max}=\max\left\{\left\lfloor \frac{{\hat{y}}_{ij}}{H}\right\rfloor ,\left\lfloor \frac{{\hat{y}}_{i+1,j}}{H}\right\rfloor ,\left\lfloor \frac{{\hat{y}}_{i,j+1}}{H}\right\rfloor ,\left\lfloor \frac{{\hat{y}}_{i+1,j+1}}{H}\right\rfloor \right\}. \end{array}$$
  Here, ⌊x⌋ is the largest integer ≤x. The definitions of $I_{\min}$ and $J_{\min}$ are similar.
Step (c): Split ${□}_{ij}$ into two triangles: ${▵}_{ij,1}$ and ${▵}_{ij,2}$ in XOY in Figure 12, where ${\hat{▵}}_{ij,\ell }$ are also triangles, and find all possible (I,J) in XOY such that
  (26) $$\begin{array}{ccc} & & \left(IH,JH\right)\in {\hat{▵}}_{ij,\ell }. \end{array}$$
  The $\lambda_{1}$, $\lambda_{2}$, and $\lambda_{3}$ are obtained from the following equations:
  $$\begin{array}{ccc} & & \left\{\begin{array}{c} x_{I}=\lambda_{1}x_{a}+\lambda_{2}x_{b}+\lambda_{3}x_{c},\\ y_{J}=\lambda_{1}y_{a}+\lambda_{2}y_{b}+\lambda_{3}y_{c},\\ 1=\lambda_{1}+\lambda_{2}+\lambda_{3}, \end{array}\right. \end{array}$$
  where a,b,c are the vertices of triangles ${\hat{▵}}_{ij,\ell },\;\ell =1,2$, and $\left(x_{a},y_{a}\right)$ are the coordinates of the vertices in Figure 12. The sufficient and necessary conditions for (26) are $0\leq \lambda_{i}\leq 1,\;i=1,2,3$.
Step (d): Obtain the approximate values of $\left(\xi_{IJ},\eta_{IJ}\right)$ related to (IH,JH) in (26) by
  $$\begin{array}{ccc} & & {\hat{\xi }}_{IJ}=\lambda_{1}\xi_{\bar{a}}+\lambda_{2}\xi_{\bar{b}}+\lambda_{3}\xi_{\bar{c}},\;\;{\hat{\eta }}_{IJ}=\lambda_{1}\eta_{\bar{a}}+\lambda_{2}\eta_{\bar{b}}+\lambda_{3}\eta_{\bar{c}}, \end{array}$$
  where $\bar{a}$, $\bar{b}$, and $\bar{c}$ are the vertices of ${▵}_{ij,\ell }$, as shown in Figure 12.

Note that Steps (a)–(d) above are easy to perform. The computation complexity is also only $O(M^{2})$, where $M^{2}$ is the total pixel number. More importantly, the computations for faces resembling under *T* and $T^{-1}$ do not involve any nonlinear solutions.

## 4. Image Experiments of Age Effects and Face Resembling

The second goal of this paper is to develop and extend the face transformations in [12]. In Figure 3, we converted the female image using the face boundary of the male. By harmonic transformations, the numerical algorithms are given in Steps I–III in Section 1.1, where the finite volume method (FVM) with Delaunay triangulation was used. An image of a teenage girl was produced and is shown in the center of Figure 3. She looks pretty, but she is virtual. No such girl exists in the world.

**Figure 13:**
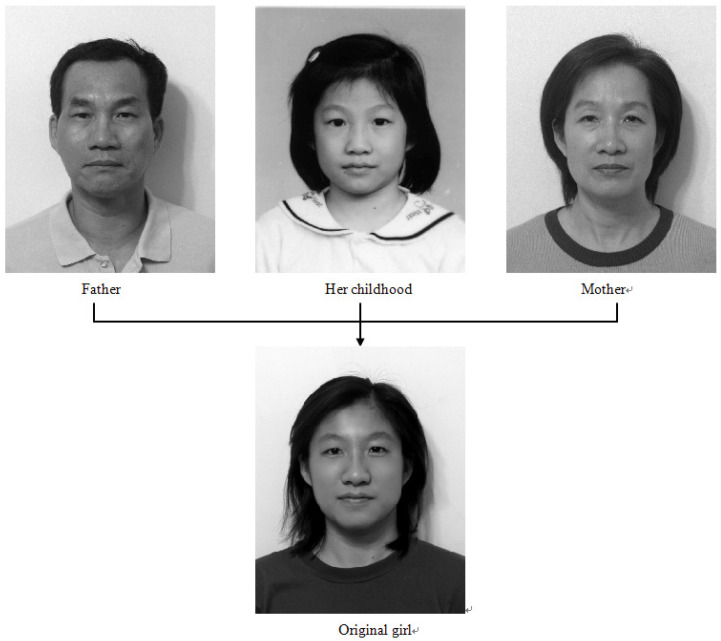
The pictures of a young girl (original girl), her father (Father), her mother (Mother), and her childhood (Child) (with the permission from C.C. Chen).

Encouraged by Figure 3, we propose a new challenging task. Based on the photos of the parents at 50 years old and the child at five years old at the top of Figure 13, we will seek the integrated image to resemble the current image of her at 20 years old at the bottom of Figure 13 as closely as possible. This face resembling is more challenging than the face transformation in [12]. Furthermore, age effects can also be found in Palumbo et al. [39] and Taskiran et al. [40]. Now, we report two image experiments.

**Figure 14:**
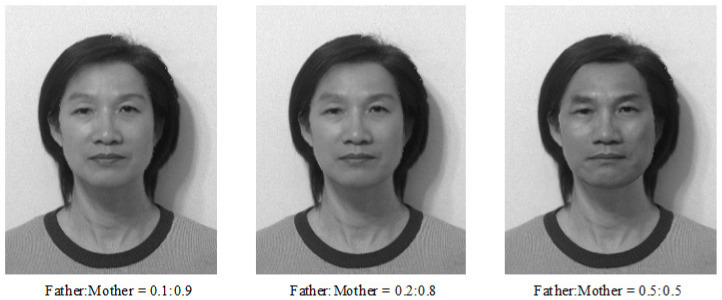
Age effects of parents’ images (with the permission from C.C. Chen).

**Figure 15:**
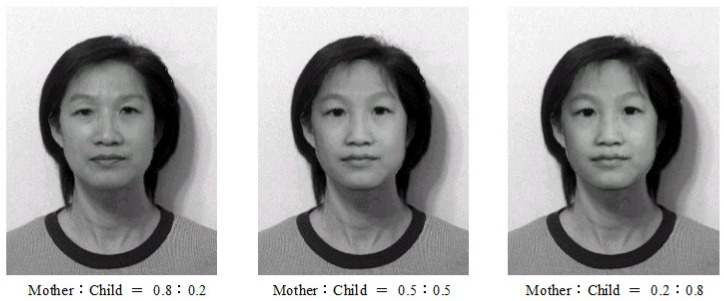
Age effects of the mother and child (with the permission from C.C. Chen).

**Figure 16:**
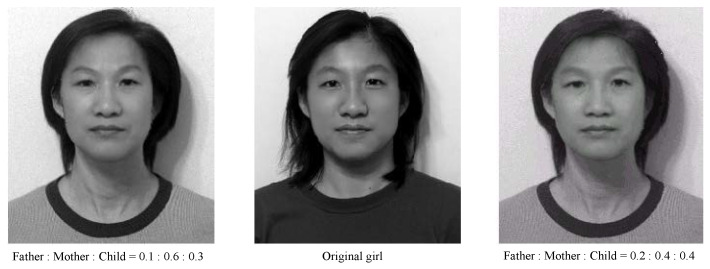
The resembling images chosen by us (with the permission from C.C. Chen).

Experiment I: Age Effects of Face Appearance. First, we choose the images of the parents only. The images of the father and mother are converted to the same frame of the young girl, and the combined images with different ratios are provided in Figure 14, where “father:mother = 0.1:0.9” denotes the proportions of grayness values given by 10% of the father’s image and 90% of the mother’s image. Disappointedly, the combined images look like a 50-year-old, not like a 20-year-old. Second, we choose images of the mother and child; the combined images are provided in Figure 15. Surprisingly, the images with different ages between teenagers and adults at 50 years old can be observed clearly.
Experiment II: Face Resembling. Based on Experiment I, we add the child’s image and choose three bases of images at the top of Figure 13 to resemble the young girl’s image. Since the young girl looks more like her mother, we may assume that the proportion (i.e., contribution) of her father is less, say about 10% and 20%. Then, we may select the resembling images among many possible combinations, which are provided in Figure 16 with their grayness proportion values. However, this selection requires a great deal of computational work. Also, the resemblance selection purely by our eyes may not be trustworthy and reasonable. New numerical techniques are required to choose optimal images automatically.

When three face images of father, mother, and child have been reshaped to the girl’s face frame (see the bottom of Figure 13), we denote their pixel grayness as $u_{ij}$, $v_{ij}$, and $w_{ij}$ at (i,j), respectively. Also, the pixel grayness of the young girl is denoted by $\phi_{ij}$. The sets of $\left\{u_{ij}\right\}$, $\left\{v_{ij}\right\}$, and $\left\{w_{ij}\right\}$ are regarded as three bases, and a linear combination of them is given by $\left\{z_{ij}\right\}=\alpha \left\{u_{ij}\right\}+\beta \left\{v_{ij}\right\}+\gamma \left\{w_{ij}\right\}$, where α, β, and γ are positive proportions to be sought. The optimal proportions α, β, and γ should be chosen to achieve $\left\{\phi_{ij}\right\}\approx \left\{z_{ij}\right\}$ as best as possible. To this end, the least squares method (LSM) may be employed. The LSM is a popular technique for geometric transformations.

Define the errors

$$\begin{array}{ccc} & & E=E\left(\alpha ,\beta ,\gamma \right)=\sum_{ij}{\left\{\phi_{ij}-z_{ij}\right\}}^{2}=\sum_{ij}{\left\{\phi_{ij}-\left(\alpha u_{ij}+\beta v_{ij}+\gamma w_{ij}\right)\right\}}^{2}. \end{array}$$

The optimal α,β,γ, satisfy the extreme conditions: $\frac{\partial E}{\partial \alpha }=0$, $\frac{\partial E}{\partial \beta }=0$, and $\frac{\partial E}{\partial \gamma }=0$. This gives a system of three linear algebraic equations:

(27) $$\begin{array}{ccc} & & a_{11}\alpha +a_{12}\beta +a_{13}\gamma =b_{1},\;\;a_{21}\alpha +a_{22}\beta +a_{23}\gamma =b_{2},\;\;a_{31}\alpha +a_{32}\beta +a_{33}\gamma =b_{3}, \end{array}$$

where the coefficients $a_{ij}=a_{ji}$, and the explicit constants are given by

$$\begin{array}{ccc} & & a_{11}=\sum_{ij}u_{ij}^{2},\;\;a_{22}=\sum_{ij}v_{ij}^{2},\;\;a_{33}=\sum_{ij}w_{ij}^{2},\\ & & a_{12}=\sum_{ij}u_{ij}v_{ij},\;\;a_{13}=\sum_{ij}u_{ij}w_{ij},\;\;a_{23}=\sum_{ij}v_{ij}w_{ij},\\ & & b_{1}=\sum_{ij}u_{ij}\phi_{ij},\;\;b_{2}=\sum_{ij}v_{ij}\phi_{ij},\;\;b_{3}=\sum_{ij}w_{ij}\phi_{ij}. \end{array}$$

**Figure 17:**
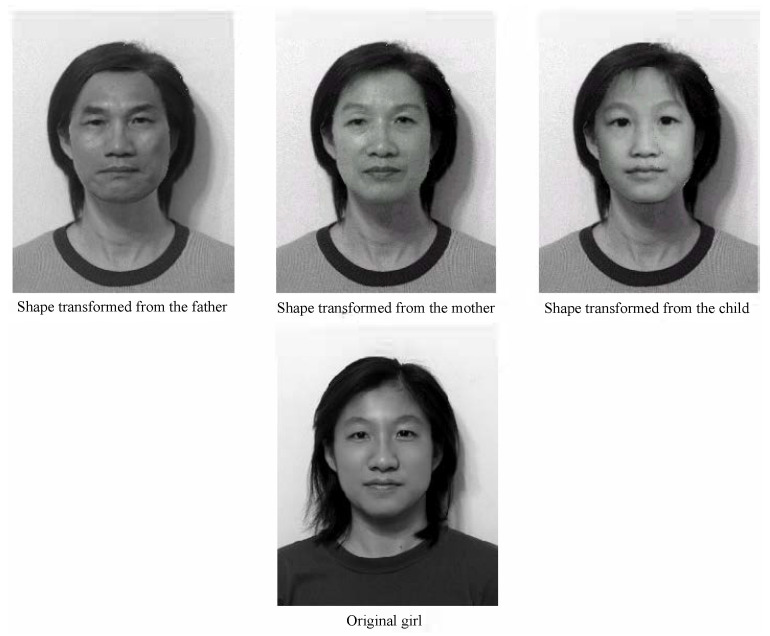
Images of father, mother, and child at the top, reshaped to the girl’s image at the bottom (with the permission from C.C. Chen).

**Figure 18:**
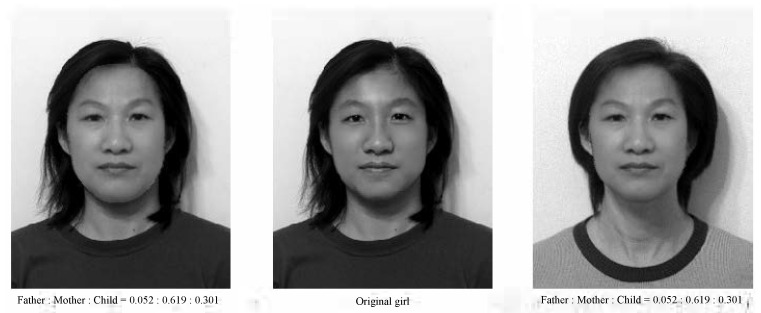
The optimal resembling images obtained by the least squares method (with the permission from C.C. Chen).

Since the total number of (i,j) is much larger than three, the linear Equation (27) are linearly independent. Hence, the values of α, β, and γ can be easily obtained and given by α=0.052, β=0.619, and γ=0.301, as shown in Figure 17, with different hairstyles. Amazingly, two artificial integrated images with such optimal proportions reach a good resemblance to the girl’s original image in the center of Figure 18.

To close this section, we introduce two remarks.

**Remark** **1.** *Face resembling in this paper is critical to finding missing children and identifying criminals via the police. Note that the face images in Figure 13 are standard photos required in a passport: shot from the front with no smile and a closed mouth. Suppose a target and potential patterns are given, where the patterns are of standard face images, but the face target suffers from geometric transformations and illumination effects since it may be taken from a cell phone by chance. The geometric transformations include basic geometric transformations (such as translation, rotation, and scaling) and prospective transformation. Our main efforts are paid to seek these geometric transformations and illumination effects. Then, the restored target can be found by the numerical algorithms in this paper, and the identification of the restored target with one of the standard patterns may be accomplished using the least squares method (LSM). Details are reported in another paper.*

**Remark** **2.** *In this remark, let us address the new techniques of face resembling proposed in this paper. The algorithms in Steps I–III in Section 1 are, indeed, of numerical interpolation, numerical integration, numerical ordinary differential equations (ODE), and numerical partial differential equations (PDE). These numerical algorithms were developed from our previous study [13,14,25] for geometric image transformations. From Figure 3, Figure 14, Figure 15, Figure 16, Figure 17 and Figure 18, the virtual face images were created by merging two (or more) face images; they belong to face morphing. Since numerical algorithms are used, numerical face resembling (or morphing) may be called. The numerical algorithms in this paper and [12] are different from those in the face recognition method in [4,8,9,10], where convolutional neural networks (CNN), textual transformations, etc., are used. Our key numerical algorithms are also different from those used in face morphing [3,5,6,11], where deep neural networks, feature comparisons, cross-dissolve techniques, etc., are used. The numerical interpolation in this paper and the Delaunay triangulation in [12] are found in [6]. Furthermore, the rapping techniques in [6] and the facial landmark techniques in [11] are also related to geometrical transformations. The advanced numerical algorithms in this paper and [12] may be used to improve the quality of face morphing. On the other hand, their effective techniques may also be employed for better identification, as in Figure 13 and Figure 16. When face morphing is used by an attacker in malice, face verification is vulnerable. Face-morphing attacks have appeared since 2014. Our numerical techniques can also be used for morphing attack detection (MAD). To describe new numerical algorithms, some computational formulas are essential. To display the efficiency of the algorithms, not only are some face images with grayness errors provided, but also the error analysis of algorithms as in [13] is needed. Nowadays, artificial intelligence (AI) is the hottest topic in research and applications since ChatGPT and Sora appeared. AI includes three basic elements: data, models, and algorithms. In face resembling (or morphing), geometrical and harmonic transformations are the models. We do not deal with massive data but seek good numerical algorithms. Hence, the CPU time can be saved greatly. In summary, the new and advanced numerical algorithms of this paper and [12] can also be applied to Sora to greatly enhance further developments of AI.*

## 5. Concluding Remarks

To conclude, let us address the novelties in this paper.

1. The face transformation achieved by harmonic models is shown in three steps in Section 1; this paper focuses on Step I to generate the face boundary, which is needed in the Dirichlet boundary condition for Laplace’s equations in harmonic models.
2. We provide two methods for blending boundary curves: the cubic spline and the ODE using the Hermite interpolation. The latter is new and developed from our previous study [17]. For the interior and exterior boundary of the face contour, the curves are piecewise smooth only with second-order derivatives. Under some constraints of characteristic points, the remaining displacements and derivatives of the curves can be determined uniquely by minimizing the energy (15).
3. The obtained curves have small curvatures, and they are smooth (or piecewise smooth). Moreover, the popular cubic spline is proved in Appendix A to be a special case of the ODE using the Hermite interpolation. A combined technique in Appendix B is then explored to couple the cubic spline and the ODE using the Hermite interpolation.
4. By the SIM, for the general nonlinear functions x(ξ,η) and y(ξ,η) in (2), their solutions are needed. However, the approximate harmonic functions are piecewise linear so that the nonlinear solutions can be bypassed. Hence, the algorithms of the SIM with μ=1 for harmonic transformations are simple, while the convergence rate $O(N^{-2})$ may remain higher than $O(N^{-1.5})$ by the SSM for *T* used.
5. Two image experiments are provided in Section 4. First, different ages of facial appearance can be produced by the images of adults and children. Then, a young boy’s/girl’s image can be resembled from his/her parents and those from their childhood.
6. Inspired by two successful experiments, many applications of face transformations may follow: modifying facial appearance, plastic surgery, generating possible offspring images for marriage counseling, and identifying a person for different purposes, such as finding missing children and confirming criminals.
7. The boundary techniques and the face combinations used in this paper can be applied not only to image processing and pattern recognition but also to face fusion and morphing, morphing attack detection (MAD), and computer animation, such as Sora, to greatly enhance further developments in AI. See Remarks 1 and 2.

## Figures and Tables

**Figure A1:**
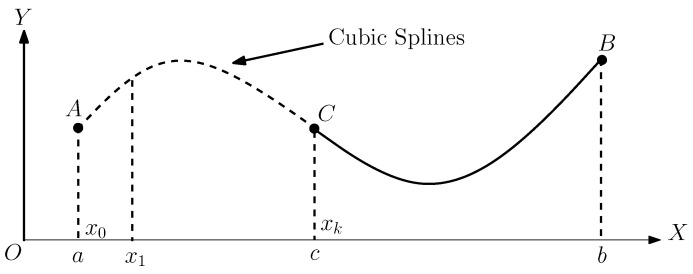
A combination of cubic splines and ODE solutions.

## Data Availability

The original contributions presented in the study are included in the article, further inquiries can be directed to the corresponding authors.

## Appendix A. Proofs for Theorem 1

In this first appendix, we will provide the direct but heuristic proofs of Theorem 1, although other proofs can be found based on Vandergraft [41] (Theorem 4.2, p. 136). We will derive the explicit equations for the Hermite function just as those of the cubic spline functions, by which we will explore the combined algorithms of the two methods given in Appendix B. First, let us derive the cubic splines from the ODE using the Hermite interpolation. For simplicity, we use the simple function y=f(x) to denote the curve since the parametric functions (x(S),y(S)) can be proved similarly.

Let $\bar{S}=\frac{x-x_{i-1}}{h_{i}}$. From [21] (p. 12), we obtain the ODE Hermite interpolation from (11) and (12),

$$\begin{array}{ccc} & & y\left(x\right)=m_{i-1}\frac{{\left(x_{i}-x\right)}^{2}\left(x-x_{i-1}\right)}{h_{i}^{2}}-m_{i}\frac{{\left(x-x_{i-1}\right)}^{2}\left(x_{i}-x\right)}{h_{i}^{2}}\\ & & +y_{i-1}\frac{{\left(x_{i}-x\right)}^{2}\left[2\left(x-x_{i-1}\right)+h_{i}\right]}{h_{i}^{3}}+y_{i}\frac{{\left(x-x_{i-1}\right)}^{2}\left[2\left(x_{i}-x\right)+h_{i}\right]}{h_{i}^{3}}. \end{array}$$

Then, we have the first- and second-order derivatives,

$$\begin{array}{ccc} & & y^{\prime }\left(x\right)=m_{i-1}\frac{\left(x_{i}-x\right)\left(2x_{i-1}+x_{i}-3x\right)}{h_{i}^{2}}\\ & & -m_{i}\frac{\left(x-x_{i-1}\right)\left(2x_{i}+x_{i-1}-3x\right)}{h_{i}^{2}}+6\frac{y_{i}-y_{i-1}}{h_{i}^{3}}\left(x_{i}-x\right)\left(x-x_{i-1}\right), \end{array}$$

(A1) $$\begin{array}{ccc} & & \;y^{\prime \prime }\left(x\right)=2m_{i-1}\frac{3x-2x_{i}-x_{i-1}}{h_{i}^{2}}+2m_{i}\frac{3x-x_{i}-2x_{i-1}}{h_{i}^{2}}+6\frac{y_{i}-y_{i-1}}{h_{i}^{3}}\left(x_{i}+x_{i-1}-2x\right). \end{array}$$

The two-sided limits of the second-order derivatives at $x_{i}$ are given by

(A2) $$\begin{array}{ccc} & & y^{\prime \prime }\left(x_{i}^{-}\right)=\frac{2m_{i-1}}{h_{i}}+\frac{4m_{i}}{h_{i}}-6\frac{y_{i}-y_{i-1}}{h_{i}^{2}}, \end{array}$$

(A3) $$\begin{array}{ccc} & & y^{\prime \prime }\left(x_{i}^{+}\right)=-\frac{4m_{i}}{h_{i+1}}-\frac{2m_{i+1}}{h_{i+1}}+6\frac{y_{i+1}-y_{i}}{h_{i+1}^{2}}. \end{array}$$

Moreover, the continuity of the second-order derivatives at an internal knot, i.e., $y^{\prime \prime }\left(x_{i}^{-}\right)=y^{\prime \prime }\left(x_{i}^{+}\right)$, yields

(A4) $$\begin{array}{ccc} & & \lambda_{i}m_{i-1}+2m_{i}+\mu_{i}m_{i+1}=c_{i},\;\;i=1,2,\ldots ,n-1, \end{array}$$

where

(A5) $$\begin{array}{ccc} & & \;\lambda_{i}=\frac{h_{i+1}}{h_{i}+h_{i+1}},\;\mu_{i}=\frac{h_{i}}{h_{i}+h_{i+1}},\;c_{i}=\frac{3}{h_{i}+h_{i+1}}\left(h_{i}\frac{y_{i+1}-y_{i}}{h_{i+1}}+h_{i+1}\frac{y_{i}-y_{i-1}}{h_{i}}\right). \end{array}$$

Equations (A4) and (A5) are the exact cubic interpolation (10). This implies that by adding the continuity of second-order derivatives at the nodes, the ODE Hermite solutions lead to the cubic spline interpolant.

On the other hand, we may also explicitly derive the equations for the minimum energy $E^{*}\left(y\left(S\right)\right)$ in (19) to provide it exactly (A4). Since p(S)≡1 and f≡0, we consider the energy $E\left(y\right)=\sum_{i=1}^{n}E_{i}\left(y\right)$, where

$$\begin{array}{ccc} & & E_{i}\left(y\right)=\frac{1}{2}\int_{x_{i-1}}^{x_{i}}{\left(y^{\prime \prime }\left(x\right)\right)}^{2}\;dx. \end{array}$$

The derivatives $y^{\prime \prime }\left(x\right)$ of order two in (A1) are simplified as

$$\begin{array}{ccc} & & y^{\prime \prime }\left(x\right)=g_{0}\left(x\right)m_{i-1}+g_{1}\left(x\right)m_{i}+g_{2}\left(x\right)\left(y_{i}-y_{i-1}\right), \end{array}$$

with the known functions

$$\begin{array}{ccc} & & g_{0}\left(x\right)=\frac{2(3x-2x_{i}-x_{i-1})}{h_{i}^{2}},\;g_{1}\left(x\right)=\frac{2(3x-x_{i}-2x_{i-1})}{h_{i}^{2}},\;g_{2}\left(x\right)=\frac{6}{h_{i}^{3}}\left(x_{i}+x_{i-1}-2x\right). \end{array}$$

Therefore, we have

(A6) $$\begin{array}{ccc} & & E_{i}\left(y\right)=\frac{1}{2}\left(m_{i-1},m_{i}\right)\left[\begin{array}{cc} a_{11} & a_{12}\\ a_{21} & a_{22} \end{array}\right]\left(\begin{array}{c} m_{i-1}\\ m_{i} \end{array}\right)+\left(m_{i-1},m_{i}\right)\left(\begin{array}{c} b_{1}\\ b_{2} \end{array}\right)+c_{0}, \end{array}$$

where $c_{0}$ is a constant, and

(A7) $$\begin{array}{ccc} & & a_{11}=\int_{x_{i-1}}^{x_{i}}{\left(g_{0}\left(x\right)\right)}^{2}\;dx,\;a_{22}=\int_{x_{i-1}}^{x_{i}}{\left(g_{1}\left(x\right)\right)}^{2}\;dx,\;a_{12}=a_{21}=\int_{x_{i-1}}^{x_{i}}g_{0}\left(x\right)g_{1}\left(x\right)\;dx,\\ & & b_{1}=\left(y_{i}-y_{i-1}\right)\int_{x_{i-1}}^{x_{i}}g_{0}\left(x\right)g_{2}\left(x\right)\;dx,\;b_{2}=\left(y_{i}-y_{i-1}\right)\int_{x_{i-1}}^{x_{i}}g_{1}\left(x\right)g_{2}\left(x\right)\;dx. \end{array}$$

Next, we evaluate the integrals (A7) by calculus. By using the transformation

$$\begin{array}{ccc} & & t=\frac{x-x_{i-1}}{h_{i}},\;\;h_{i}=x_{i}-x_{i-1},\;\;dx=h_{i}\;dt, \end{array}$$

the explicit entry $a_{11}$ is obtained by

$$\begin{array}{ccc} & & a_{11}=\int_{x_{i-1}}^{x_{i}}{\left[\frac{2(3x-2x_{i}-x_{i-1})}{h_{i}^{2}}\right]}^{2}\;dx=\frac{4}{h_{i}^{4}}\int_{0}^{1}{\left[h_{i}\left(3t-2\right)\right]}^{2}h_{i}\;dt=\frac{4}{h_{i}}. \end{array}$$

Also, we obtain other explicit entries similarly,

$$\begin{array}{ccc} & & a_{22}=\frac{4}{h_{i}^{4}}\int_{0}^{1}{\left(h_{i}\left(3t-1\right)\right)}^{2}h_{i}\;dt=\frac{4}{h_{i}},\;\;a_{12}=\frac{4}{h_{i}}\int_{0}^{1}\left(3t-1\right)\left(3t-2\right)\;dt=\frac{2}{h_{i}},\\ & & b_{1}=\frac{12}{h_{i}^{2}}\left(y_{i}-y_{i-1}\right)\int_{0}^{1}\left(3t-2\right)\left(1-2t\right)dt=-\frac{6}{h_{i}^{2}}\left(y_{i}-y_{i-1}\right),\\ & & b_{2}=\frac{12}{h_{i}^{2}}\left(y_{i}-y_{i-1}\right)\int_{0}^{1}\left(3t-1\right)\left(1-2t\right)\;dt=-\frac{6}{h_{i}^{2}}\left(y_{i}-y_{i-1}\right). \end{array}$$

Hence, the matrix and vector in (A6) are expressed explicitly,

(A8) $$\begin{array}{ccc} & & \left[\begin{array}{cc} a_{11} & a_{12}\\ a_{21} & a_{22} \end{array}\right]=\frac{2}{h_{i}}\left[\begin{array}{cc} 2 & 1\\ 1 & 2 \end{array}\right],\;\left[\begin{array}{c} b_{1}\\ b_{2} \end{array}\right]=-\frac{6(y_{i}-y_{i-1})}{h_{i}^{2}}\left[\begin{array}{c} 1\\ 1 \end{array}\right]. \end{array}$$

From the derivatives

$$\begin{array}{ccc} & & \frac{\partial }{\partial x_{i}}\left(E\left(y\right)\right)=\frac{\partial }{\partial x_{i}}\sum_{i=1}^{n}E_{i}\left(y\right)=0, \end{array}$$

we have the algebraic equations

(A9) $$\begin{array}{ccc} & & Ax=b. \end{array}$$

From (A6) and (A8), the interior equations are obtained,

$$\begin{array}{ccc} & & \frac{2}{h_{i}}\left[m_{i-1}+2m_{i}\right]-\frac{6}{h_{i}^{2}}\left(y_{i}-y_{i-1}\right)+\frac{2}{h_{i+1}}\left(2m_{i}+m_{i+1}\right)-\frac{6}{h_{i+1}^{2}}\left(y_{i+1}-y_{i}\right)=0. \end{array}$$

This is (A4) and completes the proof of Theorem 1. □

## Appendix B. Combinations of Cubic Splines and ODE Approaches

In the second Appendix, new combinations of cubic splines and ODE solutions are explored, as in Figure A1. For simplicity, we only consider y=y(x) since the case of parametric functions can be extended easily. Define an energy

$$\begin{array}{ccc} & & E\left(y\right)=\frac{1}{2}\int_{a}^{b}p\left(x\right){\left(y^{\prime \prime }\left(x\right)\right)}^{2}dx-\int_{a}^{b}f\left(x\right)y\left(x\right)\;dx, \end{array}$$

where p(x)=1 in [a,c], f(x)=0 in [a,c], with a<c<b. From Theorem 1 above, the minimum of $E^{*}\left(y\right)$ is exactly the cubic spline, where

$$\begin{array}{ccc} & & E^{*}\left(y\right)=\frac{1}{2}\int_{a}^{c}{\left(y^{\prime \prime }\left(x\right)\right)}^{2}\;dx. \end{array}$$

We conclude that the cubic spline is just a special case when p(x)≡1 and f(x)≡0. Now we may carry out a combination as in Figure A1, where point c∈(a,b) is the interior point such that $y^{\prime \prime }\left(x\right)\in C^{2}\left(a,c\right)\wedge y^{\prime }\left(x\right)\in C^{1}\left[a,b\right]$.

Now, we give the algorithms for a combination in [a,b] in Figure A1 to couple the cubic spline interpolant in (a,c) and the ODE approaches with the Hermite interpolant in (c,b). Assume $(x_{i},y_{i}),i=0,1,\ldots ,n$, $x_{k}=x_{c}$ are known, but $y_{i}^{\prime }$ are determined in the following three cases:

Case A: The equations $m_{i}\;\left(i=0,1,\ldots ,k-1\right)$ are obtained from the cubic splines in Section 2.1.
Case B: The equations $y_{i}^{\prime }=m_{i}\;\left(i=k+1,\ldots ,n\right)$ are obtained from (16) in Section 2.2.
Case C: For the equation $m_{k}$ at the intersection point *c*, there occurs the continuity of second-order derivatives. Comparing the right boundary equations in (A3) with the left boundary Equation (17), we obtain
  $$\begin{array}{ccc} & & \frac{2}{h_{k}}m_{k-1}+\left(\frac{4}{h_{k}}+{\bar{e}}_{0}\right)m_{k}+{\bar{g}}_{0}m_{k+1}+{\bar{r}}_{0}-\frac{6(y_{k}-y_{k-1})}{h_{k}}=0, \end{array}$$
  where
  $$\begin{array}{ccc} & & {\bar{e}}_{0}=\frac{2}{3h_{k+1}}\left(4p_{k}^{+}+p_{k+1}^{+}+p_{k+\frac{1}{2}}\right),\\ & & {\bar{g}}_{0}=\frac{2}{3h_{k+1}}\left(2p_{k}+2p_{k+1}-p_{k+\frac{1}{2}}\right),\;\;{\bar{r}}_{0}=-\frac{1}{12}f_{k+\frac{1}{2}}h_{k+1}^{2}. \end{array}$$

Note that by using this combination, the coefficient matrix A in (A9) is still sparse, positive definite, and symmetric. Therefore, the combined solutions can be easily obtained. It is worth pointing out that the equations in Case A are obtained from (16) in cases where $p_{k}^{-}=1,\;i\leq k$ and $f_{k}^{-}=0,\;i\leq k$.

To close this appendix, let us consider the simple support boundary condition. Suppose that the homogeneous simple support boundary condition is given by $y_{0}^{\prime \prime }=\alpha \neq 0$, and that $y_{0}$, $y_{k}$ and $y_{k}^{\prime }$ are known. We may modify the energy by

$$\begin{array}{ccc} & & E_{1}\left(y\right)=\frac{1}{2}\int_{a}^{c}{\left(y^{\prime \prime }\left(x\right)\right)}^{2}\;dx+\frac{\alpha y^{\prime }\left(a\right)}{2} \end{array}$$

or

(A10) $$\begin{array}{ccc} & & \int_{a}^{c}y^{\prime \prime }\left(x\right)w^{\prime \prime }\left(x\right)\;dx+\alpha \cdot w^{\prime }\left(a\right)=0, \end{array}$$

where w(x) are the admissible functions satisfying w(a)=w(c)=0 and $w^{\prime }\left(c\right)=0$. From integration by parts, we obtain from (A10)

$$\begin{array}{ccc} & & \int_{a}^{c}y^{\left(4\right)}w\;dx-\left(y^{\prime \prime }\left(a\right)-\alpha \right)w^{\prime }\left(a\right)=0. \end{array}$$

Since $w^{\prime }$ is arbitrary at x=a, we conclude $y^{\prime \prime }\left(a\right)=\alpha$. From (A3), we have the left boundary equation

$$\begin{array}{ccc} & & \frac{2}{h_{1}}\left[m_{1}+2m_{0}\right]-\frac{6}{h_{1}^{2}}\left(y_{1}-y_{0}\right)+\alpha =0, \end{array}$$

i.e.,

$$\begin{array}{ccc} & & 2m_{0}+m_{1}=3\left(\frac{y_{1}-y_{0}}{h_{1}}\right)-\frac{h_{1}y_{0}^{\prime \prime }}{2}, \end{array}$$

which is exactly the same as that proposed by Su and Liu [21] (p. 13).

## References

[1] Du S., Ward R.K. (2009). Improved face representation by nonuniform multilevel selection of Gabor convolution features. IEEE Trans. Syst. Man and Cybern. Part B Cybern..

[2] Chang K., Bowyer K.W., Sarkar S., Victor B. (2003). Comparison and combination of ear and face images in appearance-based biometrics. IEEE Trans. Patt. Anal. Machine Intell..

[3] Aloraibi A.Q. (2023). Image morphing techniques: A review. Technium.

[4] Indrawal D., Sharma A. (2022). Multi-module convolutional neural network based optimal face recognition with minibatch optimization. Int. J. Image Graph. Signal Process..

[5] Patel A., Lapsiwala P. (2015). Image morphing algorithm: A survey. Int. J. Comput. Appl..

[6] Scherhag U., Rathgeb C., Merkle J., Breithaupt R., Bush C. (2019). Face recognition systems under morphing attacks: A Survey. IEEE Access.

[7] Scherhag U., Rathgeb C., Merkle J., Busch C. (2020). Deep face representations for differential morphing attack detection. IEEE Trans. Inf. Forensics Secur..

[8] Tuncer T., Dogan S., Subasi A. (2023). Automated facial expression recognition using novel textural transformation. J. Ambient Intell. Human. Comput..

[9] Guo G., Zhang N. (2019). A survey on deep learning based face recognition. Comput. Vis. Image Underst..

[10] You M., Han X., Xu Y., Li L. (2020). Systematic evaluation of deep face recognition methods. Neurocomputing.

[11] Venkatesh S., Ramachandra R., Raja K., Busch C. (2021). Face morphing attack generation and detection: A comprehensive survey. IEEE Trans. Technol. Soc..

[12] Li Z.C., Chiang J.Y., Suen C.Y. (2010). Face transformation with harmonic models by finite volume method with Delaunay triangulation. IEEE Trans. Syst. Man and Cybern. Part B Cybern..

[13] Li Z.C. (1996). Analysis of discrete techniques for image transformations. Numer. Algor..

[14] Li Z.C., Bui T.D., Tang Y.Y., Suen C.Y. (1989). Computer Transformation of Digital Images and Patterns.

[15] Farin G. (1990). Curve and Surfaces for Computer Aided Geometric Design, A Practical Guide.

[16] Foley J.D., van Dam A., Feiner S.K., Hughes J.K. (1990). Computer Graphics, Principles and Practice.

[17] Li Z.C., Huang H.T. (2000). Blending curves for landing problems by numerical differential equations, II. Numerical methods. Comput. Math. Appl..

[18] Leichter I., Lindenbaum M., Rivlin E. (2009). Tacking by afffine kernel transformations using color and boundary cues. IEEE Trans. Patt. Anal. Machine Intell..

[19] Stahl J.S., Wang S. (2008). Globally optimal grouping of symmetric closed boundaries by combining boundary and region information. IEEE Trans. Patt. Anal. Machine Intell..

[20] DeCarlo D., Metaxas D. (2003). Shape evolution with structural and topological changes using blending. IEEE Trans. Patt. Anal. Machine Intell..

[21] Su B.Q., Liu D.Y. (1989). Computational Geometry Curve and Surface Modeling.

[22] Ostermann J., Pandzic I.S., Forchheimer R. (2002). Face Animation in MPEG-4. MPEG-4 Facial Animation: The Standard, Implementation and Applications.

[23] Hageman L.A., Young D.M. (1981). Applied Iteration Method.

[24] Chen C.C. (2002). Face Transformation by Harmonic Model, Generating the Face Boundary. Master’s Thesis.

[25] Li Z.C. (1998). Combined Methods for Elliptic Equations with Singularities, Interface and Infinities.

[26] Castleman K.R., Merchant F.A., Castleman K.R. (2023). Geometric transformations. Microscope Image Processing.

[27] Lakemond N., Holmberg G., Pettersson A. (2024). Digital transformation in complex systems. IEEE Trans. Eng. Manag..

[28] de la Rosa F.L., Gomez-Sirvent J.L., Sanchez-Reolid R., Morales R., Fernandez-Caballero A. (2022). Geometric transformation-based data augmentation on defect classification of segmented images of semiconductor materials using a ResNet50 convolutional neural network. Expert Syst. Appl..

[29] Luhmann T., Robson S., Kyle S., Boehm J. (2020). Close-Range Photogrammetry and 3D Imaging.

[30] Holden M. (2008). A review of geometric transformations for nonrigid body registration. IEEE Trans. Med. Imaging.

[31] Li J., Zhou S.K., Chellappa R. (2009). Appearance modeling using a geometric transform. IEEE Trans. Image Process..

[32] Ma K., Duanmu Z., Wang Z. Geometric transformation invariance image quality assessment using convolutional neural networks. Proceedings of the 2018 IEEE International Conference on Acoustics.

[33] Gao X., Lu W., Tao D., Li X. (2009). Image quality assessment based on multiscale geometric analysis. IEEE Trans. Image Process..

[34] Fang L., Shi Z., Liu Y., Li C., Pang M., Zhao E. (2023). A general geometric transformation model for linescan image registration. EURASIP J. Adv. Signal Process..

[35] Chen J., Wang L., Li X., Fang Y. Arbitrary Continuous Geometric Transformation Networks for Image Registration. Proceedings of the Advances in Neural Information Processing Systems 32 (NeurIPS 2019).

[36] Tian C., Zheng M., Zuo W., Zhang B., Zhang Y., Zhang D. (2023). Multi-stage image denoising with the wavelet transform. Patt. Recogn..

[37] You N., Han L., Zhu D., Song W. (2023). Research on image denoising in edge detection based on wavelet transform. Appl. Sci..

[38] Püspöki Z. (2016). Local Geometric Transformations in Image Analysis.

[39] Palumbo R., Adams R.B., Hess U., Kleck R.E., Zebrowitz L. (2017). Age and gender differences in facial attractiveness, but not emotion resemblance, contribute to age and gender stereotypes. Front. Psychol..

[40] Taskiran M., Kahraman N., Erdem C.E. (2020). Face recognition: Past, present and future (a review). Digit. Signal Process..

[41] Vandergraft J.S. (1983). Introduction to Numerical Computations.

